# Infliximab trough levels are associated with endoscopic healing but not with transmural healing at one year treatment with infliximab in pediatric patients with Crohn’s disease

**DOI:** 10.3389/fimmu.2023.1192827

**Published:** 2023-06-23

**Authors:** So Yoon Choi, Yiyoung Kwon, Sujin Choi, So Mi Lee, Byung-Ho Choe, Ben Kang

**Affiliations:** ^1^ Department of Pediatrics, Kosin University Gospel Hospital, Kosin University College of Medicine, Busan, Republic of Korea; ^2^ Department of Pediatrics, School of Medicine, Inha University, Incheon, Republic of Korea; ^3^ Department of Pediatrics, School of Medicine, Kyungpook National University, Daegu, Republic of Korea; ^4^ Department of Radiology, School of Medicine, Kyungpook National University, Daegu, Republic of Korea

**Keywords:** Crohn’s disease, infliximab, magnetic resonance enterography, transmural healing, trough levels

## Abstract

**Introduction:**

It is well known that infliximab (IFX) trough levels (TLs) are associated with endoscopic healing (EH) in Crohn’s disease (CD). We investigated whether IFX TLs are associated with transmural healing (TH) in pediatric patients with CD following 1-year treatment.

**Methods:**

Pediatric patients with CD treated with IFX were included in this single-center prospective study. IFX TL tests, magnetic resonance enterography (MRE), and colonoscopies were simultaneously conducted after 1-year IFX treatment. TH was defined as a wall thickness of ≤3 mm without inflammatory signs evaluated using MRE. EH was defined as a Simple Endoscopic Score for Crohn’s disease of <3 points on colonoscopy.

**Results:**

Fifty-six patients were included. EH and TH were observed in 60.7% (34/56) and 23.2% (13/56) of patients, respectively. IFX TLs were higher in patients with EH (median, 5.6 vs. 3.4 µg/mL, P = 0.002), whereas IFX TLs showed no significant difference in patients with and without TH (median, 5.4 vs. 4.7 µg/mL, P = 0.574). No significant difference was observed in EH and TH between patients whose intervals were shortened or not. Multivariate logistic regression analysis showed that IFX TLs and disease duration to IFX initiation were associated with EH (odds ratio [OR] = 1.82, P = 0.001, and OR = 0.43, P = 0.02, respectively).

**Discussion:**

In pediatric patients with CD, IFX TLs were associated with EH but not with TH. Further studies investigating long-term TH and proactive dosing based on therapeutic drug monitoring may clarify whether an association between IFX TLs and TH exists.

## 1 Introduction

Crohn’s disease (CD) is an inflammatory bowel disease that is characterized by the development of chronic lesions throughout the gastrointestinal tract due to a systemic inflammatory reaction. If inappropriately treated, strictures and fistulas can cause irreversible intestinal damage and may even require surgery, such as bowel resection and stoma formation (1, 2). However, the advent of anti-tumor necrosis factor (anti-TNF) agents, including infliximab (IFX) and adalimumab, as treatments of CD has allowed the rapid achievement and maintenance of remission in patients of all ages, including pediatric patients (3–6). This treatment has thus improved the quality of life of patients with CD. Moreover, current treatment strategies have evolved to include the administration of biologics, including anti-TNF alpha, in the early inflammatory phase of the disease before complications develop (7–9).

Beyond simple clinical remission, the treatment of CD aims to achieve deep remission. Deep remission is an evolving concept that lacks a widely mutual definition; however, it is generally considered a state of clinical remission (symptom control) with evidence of endoscopic healing (EH) (10). More recently, the concept of transmural healing (TH) is emerging. TH is evaluated by measuring bowel wall thickness and signal intensity using a noninvasive measurement tool, ultrasonography, or magnetic resonance enterography (MRE). Recently, some studies have compared EH and TH, with all suggesting that TH could be a potential treatment endpoint in CD (11–14).

IFX is an anti-TNF alpha drug used for CD treatment, for which the therapeutic trough level (TL) range (≥3 µg/mL) has been discussed in several studies (15–17). Recently, attempts to proactively evaluate TLs are undertaken to guide clinical decision-making regarding dose intensification (18, 19). Moreover, clinicians have demonstrated a relationship between IFX TL and EH, suggesting that EH occurs at concentrations of 5 µg/mL or higher in pediatric patients with CD (20). A previous study in adult CD recommended maintaining an IFX concentration of 6–10 µg/mL to reach EH (21). In the aforementioned studies, the association between EH and IFX TL has been demonstrated; however, data regarding the association between IFX TL and TH are limited. Therefore, we aimed to investigate whether IFX TLs were associated with TH in pediatric patients with CD at 1-year IFX treatment.

## 2 Methods

### 2.1 Patients and study design

This was a single-center prospective study conducted at Kyungpook National University Children’s Hospital. Participants were pediatric patients under the age of 19 and diagnosed with moderate-to-severe luminal CD requiring IFX treatment between January 2020 and December 2021. All patients were followed up for more than 1 year. CD was diagnosed following the revised Porto criteria of the European Society for Pediatric Gastroenterology, Hepatology, and Nutrition (22). Patients were classified with moderate-to-severe CD based on a Pediatric Crohn’s Disease Activity Index (PCDAI) score of 30 points or more (23).

Indications for IFX treatment included patients with relapse during maintenance treatment with immunomodulators, or top-down treatment with IFX owing to the severity of CD at the time of diagnosis. Patients who lost their response to IFX underwent interval shortening of IFX doses to 4–6 weeks. Secondary loss of response (LOR) was defined as symptomatic inflammatory relapse (PCDAI ≥10 points with elevated C-reactive protein [CRP] or calprotectin levels) and/or endoscopic or radiologically confirmed relapse following successful primary response (24). All patients underwent colonoscopy, which was inserted up to the terminal ileum, and MRE at 1 year following treatment with IFX. Patients who were 19 years or older at the initiation of treatment with IFX, used IFX for perianal CD treatment, were not available for extra blood sampling to measure IFX TLs, and failed MRE at 1 year were excluded from this study. Routine tests of IFX TLs were checked only during the 1-year study period; however, tests were also performed in those when LOR was suspected.

The primary endpoints in this study were endoscopic and transmural remission rates following IFX treatment. The secondary endpoint was evaluation of the association of IFX TLs with EH or TH. The tertiary endpoint was evaluation of factors that affect EH and TH. This study was approved by the Institutional Review Board of Kyungpook National University Chilgok Hospital and conducted in accordance with the Declaration of Helsinki.

### 2.2 Data collection

Baseline demographic data, including age at diagnosis, sex, body mass index, growth status, family history of inflammatory bowel disease, previous history of surgery associated with CD, and use of concomitant medications, were collected. Disease phenotype at diagnosis was assessed according to the Paris classification (25). Laboratory evaluation, including inflammatory markers such as erythrocyte sedimentation rate (ESR) and CRP levels, were also collected at the time of diagnosis and at 1 year following treatment. Serum IFX TLs were measured at 1 year in each sample using a commercial enzyme-linked immunosorbent assay kit (Matriks Biotechnology Co., Ltd., Ankara, Turkey).

Colonoscopy and MRE were conducted before IFX intiation and at 1 year following treatment. The severity of mucosal involvement was assessed using the Simple Endoscopic Score for Crohn’s disease (SES-CD). All MRE images were separately interpreted by two board-certified gastrointestinal radiologists. The radiologists were blinded to the clinical information and endoscopic scores of the enrolled patients. To evaluate the degree of TH, the magnetic resonance index of activity (MaRIA) score was assessed (14, 25–28).

### 2.3 Definitions

Clinical remission was defined as a PCDAI score of <10 points. Biochemical remission was defined as a CRP level of <0.3 mg/dL. SES-CD was calculated as the sum of endoscopic parameters (ulcer size, ulcerated surface, affected surface, and luminal narrowing) of the terminal ileum and each segment of the colon. EH was defined as an SES-CD of <3 points. TH was defined as a wall thickness of ≤3 mm, with the absence of ulcers, edema, enhancement, and complications on all ileocolonic segments evaluated using MRE. Complex perianal fistulas were defined as those involving the upper part of the sphincter complex (e.g., high intersphincteric, high transsphincteric, suprasphincteric, or extrasphincteric origin of the fistula tract); have multiple external openings (tracts); are associated with pain or fluctuation suggesting a perianal abscess; and/or are associated with a rectovaginal fistula or anorectal stricture (29).

### 2.4 Statistical analysis

Comparative data for continuous variables were described as median values with interquartile ranges or mean values with standard deviations. Student’s t-test, Wilcoxon’s rank-sum test, or Bonferroni t-test were used to compare continuous variables in the groups. Chi-squared test or Fisher’s exact test was used to compare categorical variables in the groups. To examine the association between EH or TH and other variables, univariate and multivariate logistic regression analyses were performed. To investigate the crude odds ratio (OR), univariate logistic regression analysis was performed. Factors with a p-value of <0.1 in the univariate analysis were included in the multivariate analysis. Results were expressed as adjusted ORs with 95% confidence intervals (CIs). A p-value of ≤0.05 was considered statistically significant. All statistical analyses were performed using R version 3.2.3.

## 3 Results

### 3.1 Baseline characteristics at diagnosis and IFX initiation

A total of 56 patients were included in this study. Investigation of the baseline characteristics at diagnosis showed a male predominance and a median diagnosis age of 14.7 years, with most patients showing L3 involvement, and approximately 80% with L4 involvement. Overall, 47 (84%) and 36 (64%) of patients were classified as having B1 behavior and perianal disease modifiers, respectively. Growth failure was observed in approximately one-fifth of patients (**Table 1**). Baseline characteristics at IFX initiation showed a median disease duration to IFX of 0.1 years, with 53 (95%) receiving concomitant azathioprine. Five patients (8.9%) had a history of prior bowel surgery before starting IFX. Three patients received ileocecectomy, and two received small bowel resection and anastomosis. The median PCDAI score was 35 points, median ESR was 46 mm/h, median CRP level was 1.5 mg/dL, median SES-CD was 15 points, and median MaRIA score was 74.2 points (**Table 1**).

**Table 1:** Baseline characteristics of study participants (*n* = 56).

At diagnosis	
Male sex, *n* (%)	37 (66.1%)
Age at diagnosis (years), median (IQR)	14.7 (13.1–16.9)
Paris classification: age	
A1a	6 (10.7%)
A1b	35 (62.5%)
A2	15 (26.8%)
Paris classification: lower GI tract involvement, *n* (%)	
L1	7 (12.5%)
L2	1 (1.8%)
L3	48 (85.7%)
Paris classification: upper GI tract involvement, *n* (%)	
None	12 (21.4%)
L4a	16 (28.6%)
L4b	16 (28.6%)
L4a + b	12 (21.4%)
Paris classification: disease behavior, *n* (%)	
B1	47 (83.9%)
B2	6 (10.7%)
B3/B2B3	3 (5.4%)
Paris classification: perianal modifier, *n* (%)	
No	20 (35.7%)
Yes	36 (64.3%)
Paris classification: growth	
G0	46 (82.1%)
G1	10 (17.9%)
First-degree family history of IBD, *n* (%)	3 (5.4%)
At IFX initiation	
Duration from diagnosis to IFX (months), median (IQR)	0.1 (0.0–0.4)
Previous bowel surgery, *n* (%)	5 (8.9%)
Age at IFX initiation (years), median (IQR)	15.0 (13.6–18.1)
Concomitant azathioprine	53 (94.6%)
PCDAI, median (IQR)	35 (30–37.5)
WBC (/µL), median (IQR)	8,500 (7,295–10,090)
Hematocrit (%), median (IQR)	37.4 (34.2–40.0)
Platelet count (×103/μL), median (IQR)	357 (324–448)
Albumin (g/dL), median (IQR)	4.0 (3.5–4.3)
ESR (mm/h), median (IQR)	46 (33–73)
CRP (mg/dL), median (IQR)	1.46 (0.73–3.15)
FC (mg/kg), median (IQR) (*n* = 34)	2,056 (987–2,632)
SES-CD, median (IQR)	15 (11–21)
MaRIA, median (IQR)	74.2 (51.2–95.6)

IQR, interquartile range; GI, gastrointestinal; IBD, inflammatory bowel disease; L1, distal 1/3 ileum ± limited cecal disease; L2, colonic disease; L3, ileocolonic disease; L4a, upper disease proximal to the ligament of Treitz; L4b, upper disease distal to the ligament of Treitz and proximal to the distal 1/3 ileum; L4a + b, upper disease involvement in both L4a and L4b; B1, nonstricturing nonpenetrating behavior; B2, stricturing behavior; B3, penetrating behavior; IFX, infliximab; PCDAI, Pediatric Crohn’s Disease Activity Index; SD, standard deviation; SES-CD, Simple Endoscopic Score for Crohn’s Disease; WBC, white blood cell count; ESR, erythrocyte sedimentation rate; CRP, C-reactive protein; FC, fecal calprotectin; MaRIA, magnetic resonance index of activity.

### 3.2 Remission rates at 1-year treatment with IFX

**Figure 1:**
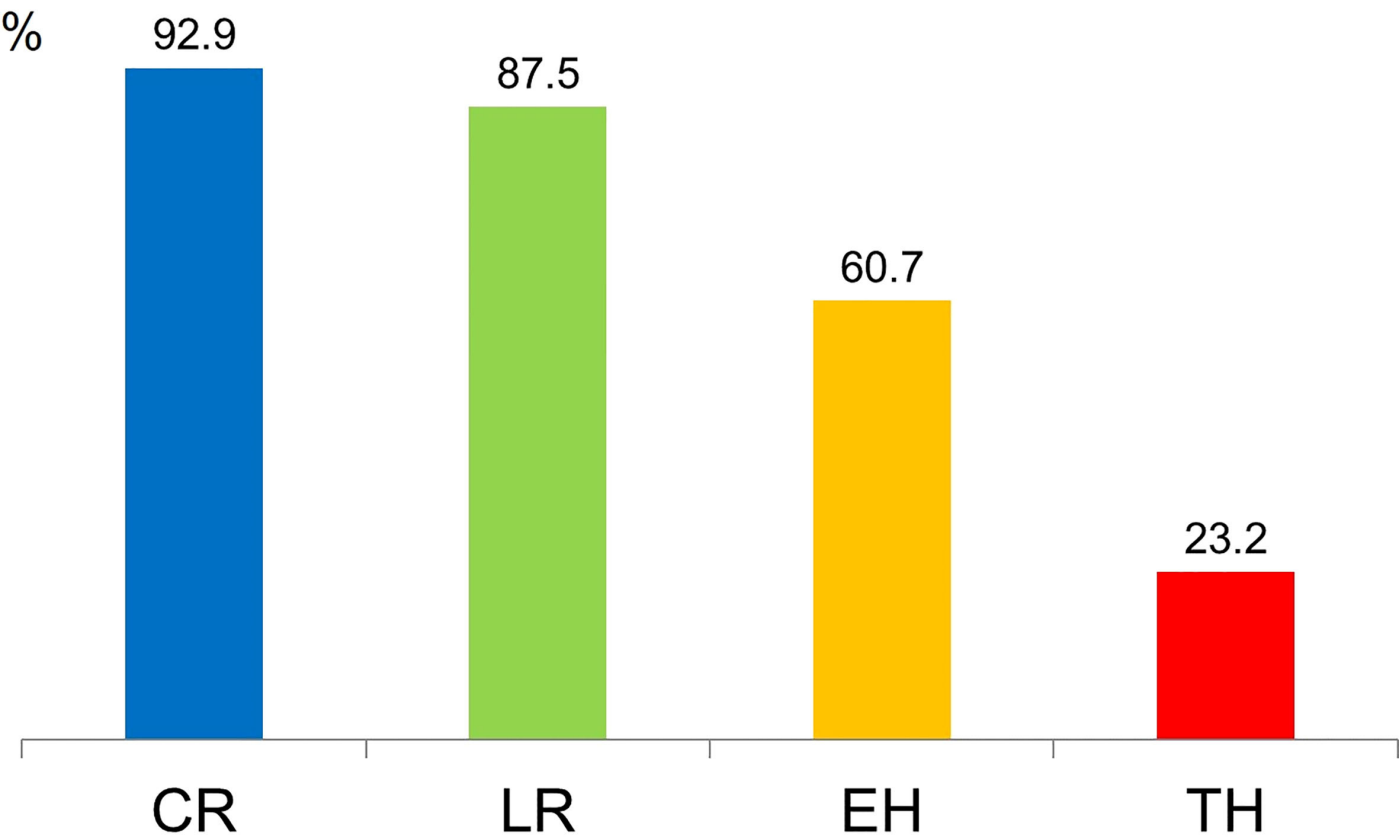
Remission rates following 1-year treatment with infliximab. CR, clinical remission; LR, laboratory remission; EH, endoscopic healing; TH, transmural healing.

Remission rates at 1-year treatment with IFX showed that 92.9% (52/56) of patients were in clinical remission, and 87.5% (49/56) had a CRP level of <0.3 mg/dL. Furthermore, EH and TH were observed in 60.7% (34/56) and 23.2% (13/56) of patients, respectively (**Figure 1**).

### 3.3 Comparison between patients with and without TH

Comparison between patients with and without TH at 1-year IFX treatment showed no significant differences at baseline (**Table 2**). However, at 1-year IFX treatment, the albumin level was significantly higher in patients with TH (4.8 ± 0.3 vs. 4.6 ± 0.3 g/dL, *P* = 0.022), whereas SES-CD (median, 0 vs. 3 points, *P* = 0.014) and MaRIA (median, 31.5 [29.7–33.8] vs. 43.9 [38.3–56.4] points, *P* < 0.001) scores were significantly lower. Furthermore, all 13 patients who achieved TH had EH. Since the definition of EH was an SES-CD of 0–2 points, the SES-CD of 13 patients who achieved TH was further analyzed. Of the 13 patients, 10, 2, and 1 scored 0, 1, and 2 points, respectively, showing that 76.9% of the patients who reached TH had an SES-CD of 0 points.

**Table 2:** Comparison of patients with and without transmural healing at 1-year IFX treatment.

Variables	Yes (n = 13)	No (n = 43)	*P*
At diagnosis and IFX initiation			
Male, *n* (%)	9 (69.2)	28 (65.1)	1.000
Age at diagnosis, *years*	14.5 ± 2.3	14.5 ± 3.1	0.967
Disease duration to IFX, *years*	0.1 (0.0–0.5)	0.0 (0.0–0.5)	0.605
Age at IFX initiation, *years*	13.9 (13.1–15.3)	15.1 (13.7–18.2)	0.183
B1 behavior at IFX initiation, *n* (%)	12 (92.3)	35 (81.4)	0.612
PCDAI at IFX initiation	32.5 (30–37.5)	35 (30–40)	0.597
CRP at IFX initiation, *mg/dL*	1.47 (0.87–2.43)	1.46 (0.73–3.62)	0.479
Albumin at IFX initiation, *g/dL*	4.0 (3.7–4.3)	3.9 (3.5–4.3)	0.815
FC at IFX initiation, *mg/kg* (*n* = 34)	1,588 (798–2,340)	2,056 (1,078–3,250)	0.519
SES-CD at IFX initiation	14.3 ± 8.4	16.8 ± 7.5	0.309
MaRIA at IFX initiation	60.1 (49.5–83.7)	75.6 (51.7–100.2)	0.277
At 1-year IFX treatment			
PCDAI	0 (0–5)	0 (0–5)	0.761
CRP, *mg/dL*	0.03 (0.03–0.04)	0.03 (0.03–0.08)	0.598
Albumin, *g/dL*	4.8 ± 0.3	4.6 ± 0.3	0.022
FC, *mg/kg* (*n* = 38)	32 (28–74)	219 (116–603)	0.001
SES-CD	0 (0–1)	3 (0–5)	0.014
MaRIA	31.5 (29.7–33.8)	43.9 (38.3–56.4)	<0.001
Concomitant azathioprine, *n* (%)	12 (92.3)	41 (95.3)	1.000
IFX TL, ***µg*** */mL*	5.4 (4.0–6.0)	4.7 (3.3–6.7)	0.574
IFX interval shortening, *n* (%)	0 (0)	12 (27.9)	0.574

IFX, infliximab; B1, nonstricturing nonpenetrating behavior; PCDAI, Pediatric Crohn’s Disease Activity Index; WBC, white blood cell; ESR, erythrocyte sedimentation rate; CRP, C-reactive protein; FC, fecal calprotectin; SES-CD, Simple Endoscopic Score for Crohn’s Disease; MaRIA, magnetic resonance index of activity; IFX TL, infliximab trough level.

### 3.4 IFX TLs according to remission status and dosing intervals

**Figure 2:**
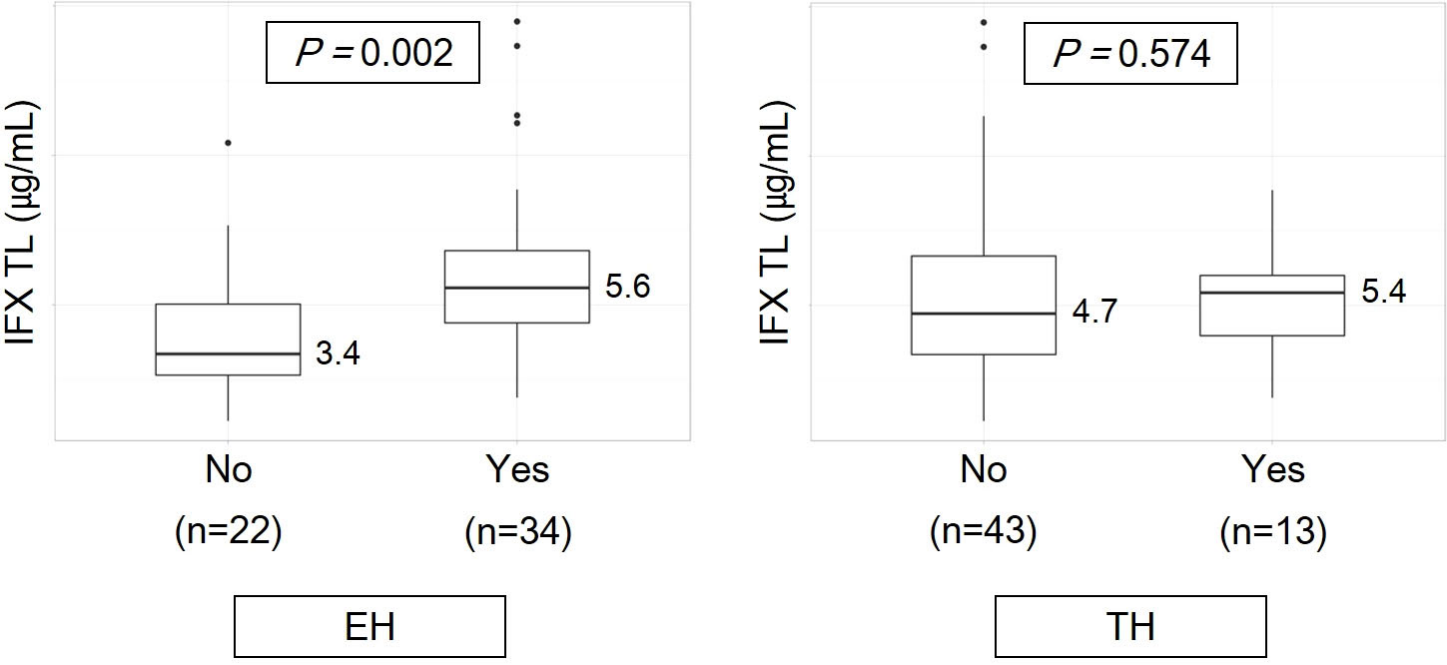
Infliximab trough levels according to remission status. EH, endoscopic healing; IFX TL, infliximab trough level; TH, transmural healing.

**Figure 3:**
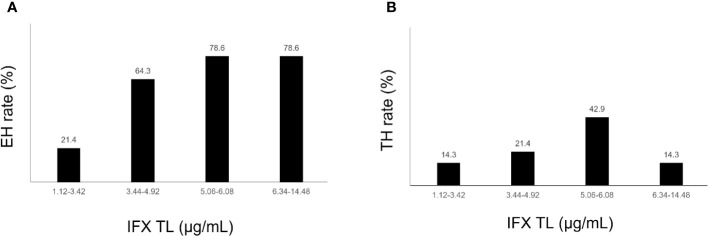
**(A)** Endoscopic healing rates according to quartiles of infliximab trough levels. **(B)** Transmural healing rates according to quartiles of infliximab trough levels. EH, endoscopic healing; IFX TL, infliximab trough level; TH, transmural healing.

**Figure 4:**
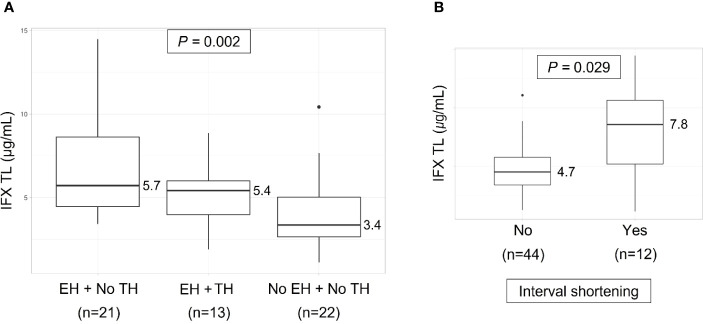
**(A)** Comparison of infliximab trough levels between the EH + TH, EH + no TH, and no EH + no TH groups. **(B)** Infliximab trough levels according to dosing intervals. EH, endoscopic healing; IFX TL, infliximab trough level; TH, transmural healing.

The comparison of IFX TLs according to remission status showed that patients with EH had significantly higher IFX TLs than those without EH (median, 5.6 vs. 3.4 μg/mL, *P* = 0.002), whereas no statistically significant difference was observed between those with and without TH (median, 5.4 vs. 4.7 μg/mL, *P* = 0.574) (**Figure 2**). The quartiles of IFX TL and proportion of patients with EH and TH are described in **Figure 3**. When IFX TLs were compared between the EH + TH, EH + no TH, and no EH + no TH groups, the median values of IFX TLs between the EH + TH and EH + no TH groups were 5.7 and 5.4 μg/mL, respectively, with no significant difference. However, in the case of the no EH + no TH group, IFX TLs were statistically significantly lower with median TLs of 3.4 μg/mL (*P* = 0.002) than the other two groups (**Figure 4A**). As all patients who had reached TH also had achieved EH, there were no patients who had only TH without EH (TH + no EH). The dosing intervals of IFX were shortened in 12 patients during maintenance. The comparison of IFX TLs according to interval shortening showed significantly higher levels in patients whose intervals were shortened (median, 7.8 vs. 4.7 μg/mL, *P* = 0.029) (**Figure 4B**). Antibodies to IFX were detected in 2 of 12 patients with shortened dosing intervals.

### 3.5 Remission rates according to dosing intervals

**Figure 5:**
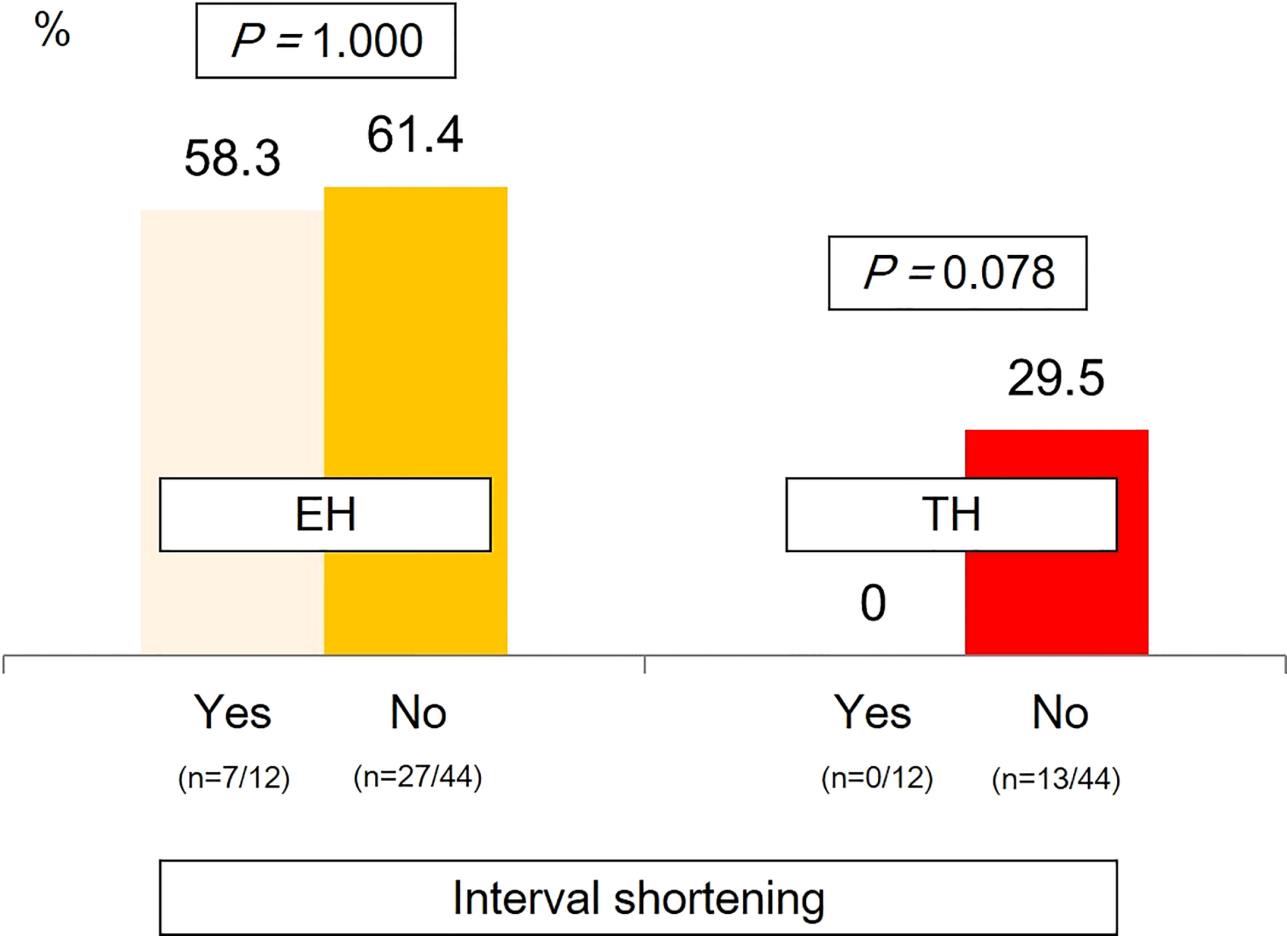
Remission rates according to dosing intervals. EH, endoscopic healing; TH, transmural healing.

Remission rates according to dosing intervals showed that the rates of EH were similar between patients whose intervals were and were not shortened. Meanwhile, those whose intervals were shortened had a relatively lower proportion of patients with TH than those receiving IFX with the standard dosing interval of 8 weeks, although statistical significance was not observed (**Figure 5**).

### 3.6 Factors associated with TH and EH at 1-year IFX treatment

In univariate analysis, albumin level (OR, 10.16; 95% CI, 1.37–92.62; *P* = 0.028) and SES-CD (OR, 0.60; 95% CI, 0.32–0.89; *P* = 0.046) at 1-year IFX treatment were significantly associated with TH. However, these associations were not significant in multivariate analysis (**Table 3**). Meanwhile, disease duration to IFX (OR, 0.43; 95% CI, 0.18–0.80; *P* = 0.023), MaRIA score at IFX initiation (OR, 0.97; 95% CI, 0.94–1.00; *P* = 0.046), and IFX TLs (OR, 1.76; 95% CI, 1.30–2.68; *P* = 0.002) were associated with EH at 1-year IFX treatment (**Table 4**).

**Table 3:** Logistic regression analyses of factors associated with transmural healing at 1-year IFX treatment.

	Univariate logistic regression analysis			Multivariate analysis with stepwise selection		
Variables	OR	95% CI	*p*	OR	95% CI	*p*
Male sex	1.21	0.33–5.05	0.784			
Age at diagnosis, *years*	1.00	0.81–1.25	0.967			
Disease duration to IFX, *years*	0.32	0.01–0.90	0.244			
Age at IFX initiation, *years*	0.91	0.73–1.12	0.379			
PCDAI at IFX initiation	0.98	0.92–1.04	0.628			
CRP at IFX initiation, *mg/dL*	0.78	0.49–1.04	0.178			
Albumin at IFX initiation, *g/dL*	1.06	0.37–3.45	0.913			
SES-CD at IFX initiation	0.96	0.87–1.04	0.305			
MaRIA at IFX initiation	0.07	0.00–2.18	0.159			
PCDAI at 1 year	0.93	0.74–1.08	0.416			
CRP at 1 year, *mg/dL*	0.03	0.00–1.18	0.324			
Albumin at 1 year, *g/dL*	10.16	1.37–92.62	0.028	7.15	0.82–79.32	0.086
SES-CD at 1 year	0.60	0.32–0.89	0.046	0.60	0.32–0.91	0.060
Concomitant azathioprine	0.59	0.05–13.23	0.673			
IFX TLs, *μg/dL*	0.96	0.75–1.19	0.736			

IFX, infliximab; PCDAI, Pediatric Crohn’s Disease Activity Index; CRP, C-reactive protein; SES-CD, Simple Endoscopic Score for Crohn’s Disease; MaRIA, magnetic resonance index of activity; IFX TL, infliximab trough level; OR, odds ratio; CI, confidence interval.

**Table 4:** Logistic regression analyses of factors associated with mucosal healing at 1-year IFX treatment.

	Univariate logistic regression analysis			Multivariate analysis with stepwise selection		
	OR	95% CI	*p*	OR	95% CI	*p*
Male sex	0.61	0.18–1.90	0.399			
Age at diagnosis, *years*	0.93	0.77–1.12	0.482			
Disease duration to IFX, *years*	0.72	0.48–1.04	0.093	0.43	0.18–0.80	0.023
Age at IFX initiation, *years*	0.85	0.69–1.03	0.117			
B1 behavior at IFX initiation	3.88	0.90–20.34	0.079	4.23	0.50–49.25	0.202
PCDAI at IFX initiation	1.04	0.99–1.11	0.167			
CRP at IFX initiation, *mg/dL*	1.00	0.83–1.22	0.973			
Albumin at IFX initiation, *g/dL*	0.83	0.31–2.13	0.706			
SES-CD at IFX initiation	0.98	0.92–1.06	0.670			
MaRIA at IFX initiation	0.98	0.96–1.00	0.049	0.97	0.94–1.00	0.046
PCDAI at 1 year	0.87	0.73–0.99	0.058	0.85	0.64–1.03	0.177
CRP at 1 year, *mg/dL*	0.76	0.26–1.91	0.546			
Albumin at 1 year, *g/dL*	4.29	0.72–32.11	0.126			
Concomitant azathioprine	0.59	0.05–13.23	0.673			
IFX TLs, *μg/dL*	1.51	1.15–2.15	0.010	1.76	1.30–2.68	0.002
IFX interval shortening	0.88	0.24–3.40	0.849			

IFX, infliximab; B1, nonstricturing nonpenetrating behavior; PCDAI, Pediatric Crohn’s Disease Activity Index; CRP, C-reactive protein; SES-CD, Simple Endoscopic Score for Crohn’s Disease; MaRIA, magnetic resonance index of activity; IFX TL, infliximab trough level; OR, odds ratio; CI, confidence interval.

## 4 Discussion

This study prospectively evaluated whether IFX TLs were associated with TH in pediatric patients with CD. As several recent studies have argued that TH is a promising new treatment target in CD, evaluating the relationship between IFX TLs and TH is necessary to develop new therapeutic approaches.

Consistent with previous studies, we observed a significant difference in IFX TLs between the two groups of patients with and without EH (20, 21, 30, 31); however, no difference in IFX TLs was observed between the two groups of patients with and without TH (**Figure 2**). Consequently, the authors considered TH as a deeper target for remission than EH, as patients with TH may be a subgroup of patients with EH. Unlike ulcerative colitis (UC), patients with CD exhibit deep ulcers rather than superficial ulcers; therefore, although the surface of the ulcer is healed, some inflammatory responses inside the mucosa or bowel wall (e.g., increase in blood flow and recruitment of immune cells) may yet remain.

IFX, an anti-TNF alpha agent, blocks the cytokine downstream of the inflammation process of CD; however, it does not inhibit a series of reactions that increase blood flow, recruit inflammatory cells (monocytes and macrophages), and differentiate T helper cells, which are upstream of the inflammatory process (32–34). Considering this mechanism, IFX treatment alone may prevent direct mucosal injury but not all inflammatory signaling pathways. This presents the possibility that EH can be achieved but TH cannot be achieved. In this regard, treatments other than IFX may be required to reach TH.

An additional point to consider is whether an IFX TL above the known therapeutic range is needed to obtain an association between TH and IFX TLs. This is because past studies have reported that higher IFX TL concentrations are required to treat fistulizing perianal lesions, and recent studies have evaluated the need for high-dose IFX treatment before LOR develops (35–37). However, evidence for higher IFX concentrations and doses to achieve TH remain insufficient to date. To verify this, additional studies are needed.

The third possible reason that IFX TLs were not associated with TH is that the 1-year treatment period set in this study may be relatively short to achieve TH. In 2013, Van Assche et al. reported the results of MRE up to 6 months following IFX treatment, and only 13% of patients showed normalization of MRE findings, leading them to conclude that the 6-month treatment period was insufficient for observing the treatment effect (38). However, according to the study of Castiglione et al. in 2016, the TH rate evaluated at 2 years following IFX treatment increased to 23% (39).

The fourth reason is minor but may nevertheless affect the outcome. There is no standardized definition of TH to date. In 2021, Geyl et al. compiled 17 papers that evaluated TH in a systematic review (40); the method for defining TH in each study was summarized, showing that all studies used bowel wall thickening, MaRIA score, or SES-CD. Similar to our study, most studies defined TH using bowel wall thickness; however, the standard for thickness varied, with some studies setting the upper limit as 3 mm and others as 7 mm (12, 41–51). Compared with other studies, our study’s TH definition was relatively strict. These stringent criteria may have resulted in an underestimation of the number of patients who reached TH, which may have prevented us from obtaining statistically significant results.

The results of the logistic regression analysis (**Tables 3**, **4**) support the aforementioned results. As mucosal defects result in a loss of albumin due to leakage, a high albumin level can be used as a predictor of TH (**Table 3**). Furthermore, as the SES-CD represents EH, and EH can be observed as a preceding remission of TH, patients who reach EH may have a higher probability of reaching TH. Therefore, the SES-CD can be used as a predictor of TH. As shown in the results of the correlation between EH and IFX TLs, a high IFX TL could predict EH in the logistic regression analysis (**Table 4**). Moreover, logistic regression analysis showed that achieving EH is difficult since the period from the time of diagnosis to the use of IFX increases. Consistently, previous studies have reported that rapid application of biologics results in a high remission rate (52–54).

The strength of this study is that it is the first to evaluate the relationship between IFX TLs and TH in pediatric patients with CD. Even when expanding this scope to include studies in adults, only one study has evaluated the association between IFX TLs and TH to date. This paper was published very recently, in 2022 (55), and evaluated TH using ultrasonography, not MRE, and suggested that lower maintenance IFX TLs are associated with sonographic parameters of inflammation in UC and CD. Similar to our study, this paper also states that further studies are required to determine whether targeting higher IFX TLs can increase sonographic TH. The primary limitation of this study is that only 13 patients reached TH, which was too low to reach statistical significance. However, the total number of patients, 56, was relatively large for a prospective study with pediatric patients. Furthermore, this study is the first to evaluate the relationship between IFX TLs and TH in pediatric patients.

## 5 Conclusion

The results of our analysis showed that higher IFX TLs were associated with EH but not with TH in pediatric patients with CD treated with IFX. TH should be considered a concept of deeper remission than EH, and higher TLs than conventional therapeutic IFX TLs or long-term treatment may be required to reach TH. The results of this study may serve as a basis for further studies investigating long-term TH and proactive dosing based on therapeutic drug monitoring, which may clarify whether an association between IFX TLs and TH exists.

## Data availability statement

The raw data supporting the conclusions of this article will be made available by the authors, without undue reservation.

## Ethics statement

This study was approved by the Institutional Review Board of Kyungpook National University Chilgok Hospital. Written informed consent to participate in this study was provided by the participant’ legal guardian/next of kin.

## Author contributions

SYC contributed in the analysis and interpretation of data, drafting of the initial manuscript, and critical revision for important intellectual content. YK contributed in the analysis and interpretation of data, and drafting of the initial manuscript. SJC contributed in acquisition of data, and drafting of the initial manuscript. SL contributed in in the analysis and interpretation of data, and critical revision for important intellectual content. B-HC contributed in acquisition, analysis and interpretation of data, and critical revision for important intellectual content. BK contributed in the conception and design of the study, acquisition, analysis and interpretation of data, statistical analysis, and critical revision for important intellectual content. All authors contributed to the article and approved the submitted version.
